# Type of tea consumption and depressive symptoms in Chinese older adults

**DOI:** 10.1186/s12877-021-02203-z

**Published:** 2021-05-24

**Authors:** Yao Yao, Huashuai Chen, Lele Chen, Sang-Yhun Ju, Huazhen Yang, Yi Zeng, Danan Gu, Tze Pin Ng

**Affiliations:** 1 Center for Healthy Aging and Development Studies, Raissun Institute for Advanced Studies, National School of Development, Peking University, Beijing, 100871 China; 2 Center for the Study of Aging and Human Development and Geriatrics Division, Medical School of Duke University, Durham, NC 27705 USA; 3 School of Social and Behavioral Sciences, Nanjing University, Nanjing, 100191 China; 4 Department of Family Medicine, St. Mary’s Hospital, College of Medicine, The Catholic University of Korea, Gyeonggi-do, 11765 Republic of Korea; 5 West China Biomedical Big Data Center, West China Hospital, Sichuan University, Chengdu, 610041 China; 6 Independent Researcher, New York, NY 10017 USA; 7 Department of Psychological Medicine, Yong Loo Lin School of Medicine, National University of Singapore, Singapore, Singapore

**Keywords:** Type of tea intake, Mental health, Depressive symptoms, Older adults, CLHLS

## Abstract

**Background:**

Existing research indicates that tea drinking may exert beneficiary effects on mental health. However, associations between different types of tea intake and mental health such as depression have not been fully examined. The purpose of this study was to examine the associations of green tea, fermented tea, and floral tea consumption with depressive symptoms.

**Methods:**

We used data from the 2018 wave of the Chinese Longitudinal Healthy Longevity Survey, a nationwide survey on older adults in mainland China. A total of 13,115 participants (mean age 83.7 years, 54.2% were women) with valid responses were included in the analysis. The type (green, fermented [black, Oolong, white, yellow, dark, and compressed teas], and floral) and the frequency of tea consumption were recorded, and depressive symptoms were assessed using 10-item of the Center for Epidemiologic Studies Depression Scale (CES-D-10). We examined the associations between the type and the frequency of tea intake and depression, controlling for a set of demographic, socioeconomic, psychosocial, behavioral, and health-related variables.

**Results:**

Overall, intakes of green tea, fermented tea, and floral tea were all significantly associated with lower prevalence of depressive symptoms, independent of other risk factors. Compared with the group of no tea intake, the adjusted ORs of depressive symptoms for daily green tea, fermented tea, and floral tea intake were 0.85 (95% CI: 0.76–0.95), 0.87 (95% CI: 0.76–0.99), and 0.70 (95% CI: 0.59–0.82), respectively. Linear associations were observed between the frequencies of all three types of tea intake and depressive symptoms (*P* < 0.05 for trends for all three types). The associations of the type and the frequency of tea intake and depressive symptoms were robust in several sensitivity analyses.

**Conclusions:**

Among Chinese older adults, regularly consumed any type of tea (green, fermented, or floral) were less likely to show depressive symptoms, the associations seemed more pronounced among floral tea and green tea drinkers.

**Supplementary Information:**

The online version contains supplementary material available at 10.1186/s12877-021-02203-z.

## Background

Depression is a serious mental and public health problem among older adults [1, 2]. Depression is the leading cause of disability worldwide, accounting for 5.7% of years lived with disability among the older adults [2]. Depression is underdiagnosed and undertreated [1], especially for those who were undergoing adverse events such as COVID-19 pandemic [3]. Given the increased risk of disability and mortality associated with depression [4, 5], it is vitally important to consider preventive interventions that improve the health and quality of life of older adults and reduce the burden on families and societies. Potentially useful interventions that prevent depression include cognitive and behavioral interventions, such as mindfulness-based therapy and lifestyle interventions [6, 7]. Recent research has also drawn attention to the potential anti-depressant effect of regular tea consumption [8].

Human trials, mouse models, and in vitro experiments have explored the underlying mechanisms for the neuroprotective effect and invigorating quality of tea. In mice models, green tea polyphenols exert antidepressant-like effects by inhibition of the hypothalamic–pituitary–adrenal axis [9]. Green tea catechins, such as epigallocatechin gallate (ECCG), also exert anti-inflammatory and neuroprotective actions in laboratory experiments [10]. Anti-inflammatory properties of flavonoids from green tea are also found to be associated with a lower risk of depression [11, 12]. Clinical trials show that L-theanine, a unique component in green tea, can ameliorate the stress-related symptoms and depressive disorders [13]. Evidence from human electroencephalograph (EEG) studies shows that L-theanine significantly increases activity in the alpha frequency band which indicates mental relaxation [14]. In sum, these observations support the argument that tea consumption has benefits in relaxing mood and preventing depression.

However, to date, epidemiological evidence for the beneficial effect of tea consumption in reducing the risk and the severity of depression in humans is inconclusive. Mixed findings are reported from less than a dozen cross-sectional and prospective cohort studies [15–18] and two meta-analyses [19, 20] published in 2015 and 2016 have summarized the heterogeneous association between tea drinking and depression with divergent conclusions. Since then, there have been several more cross-sectional studies that support an inverse association of tea consumption with depression [21–26]. However, there remains a paucity of studies that examine the dose-response effect for different types of tea. Bioactive components, which vary in different types of tea through diverse processing methods in the markets, may account for the heterogeneity of findings [27, 28]. Green tea has a higher content of catechins than fermented teas (such as Oolong and black teas). The fermentation process during tea manufacturing reduces the levels of catechins but elevates levels of gallic acid, theaflavins and thearubigins [29]. There is a great heterogeneity of types of tea produced that are available and consumed around the world and particularly in China where tea drinking originated.

In this study, we analyzed nationwide data of a large nationally representative sample of older adults which identified several different types of tea consumption (green tea, fermented tea, and floral tea) widely distributed geographically across China among the tea drinkers. We aimed to examine the associations between the types and the frequencies of tea consumption and depressive symptoms among older population, we additionally conducted stratified analyses by sex, age, and geographic regions to examine the heterogeneity of the associations.

## Methods

### Study population

The eighth wave of the Chinese Longitudinal Healthy Longevity Survey (CLHLS) in 2018/2019 (briefly as 2018) was used to fulfill our research goals. The CLHLS was conducted in more than 630 counties/cities in 22 provinces of 31 provinces in mainland China plus on county in Hainan Province. The study sampled provinces roughly represents about 85% of the Chinese population in 2020. Given its research focus on longevity, the CLHLS oversampled long-lived populations. For example, in the sampled counties, for every 3 centenarians, 4 participants aged 80–89, 4 participants aged 90–99, and 5 participants aged 65–79 were recruited based on predesignated age and sex. If there was no such matched person, a person will be recruited in neighboring county with same predesignated age and sex. In other words, the CLHLS oversampled centenarians and the oldest-old (aged 80 years or older). Detailed information of the CLHLS can be found elsewhere [30, 31].

**Fig. 1:**
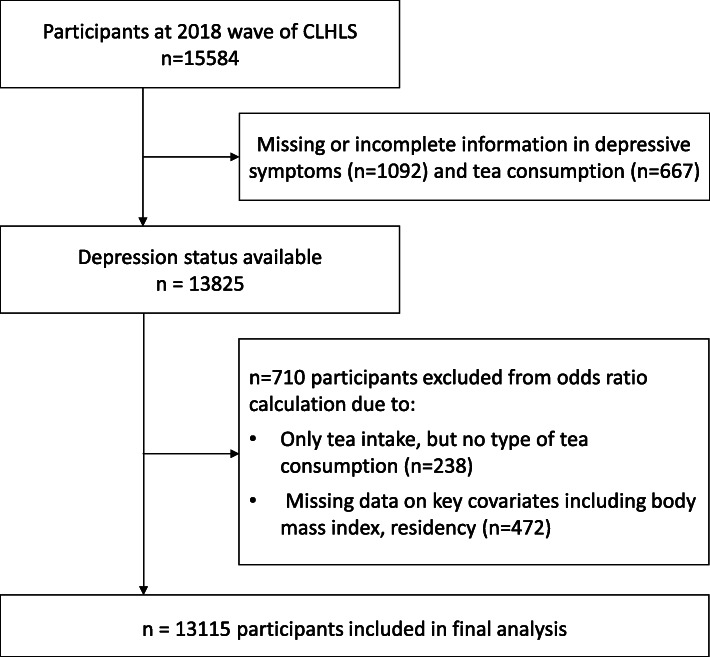
Derivation of the study population from participants of the Chinese Longitudinal Healthy Longevity Survey (CLHLS)

The 2018 wave of the CLHLS collected the self-reported types and frequencies of tea intake and assessed depressive symptoms by the Center for Epidemiologic Studies Depression Scale. After excluding 2469 participants with missing data on depressive symptoms, self-reported types of tea consumption, key covariables, the final analytical sample used in this study included 13,115 adults aged 65 years or older (5121 were aged 65–79, 6301 were aged 80–99, and 1693 were aged 100 years or older) (Fig. 1).

The Biomedical Ethics Committee, Peking University (IRB00001052–13074) approved the CLHLS. All participants or their legal representatives signed written consent forms to participate in the baseline and follow-up surveys.

### Measurements

The questionnaire in the 2018 wave of the CLHLS included items about the frequency of habitual consumption of 8 types of tea (green, black, Oolong, white, yellow, dark, compressed, and floral teas). The detailed types and classifications of tea consumption in this study are provided in supplements (Supplementary Table 1). In brief, we classified the type of tea into green tea, fermented tea (black, Oolong, white, yellow, dark, and compressed teas), and flower tea [32]. We grouped the frequency of tea consumption of each type of tea into 3 categories: daily (≥ 1 cup/day), occasionally (< 1 cup/day but ≥1 cup/month), and never or rarely (< 1 cup/month or never drink tea) [33].

We used the 10-item of the Center for Epidemiologic Studies Depression Scale (CES-D-10) to measure depressive symptoms in this study [34]. The answers were indicated in a four-scale metric, from “rarely” to “some days” (1–2 days), “occasionally” (3–4 days), or “most of the time” (5–7 days). For the two positive questions— “I was happy” and “I felt hopeful about the future”—answers were reversely coded before summation. We then coded all answers from 0 to 3 as “rarely” to “most of the time”, respectively. The total range of CES-D-10 scores in this study was 0–30, with higher scores indicating a greater severity of depressive symptoms. A person is considered to have depressive symptoms if he/she scored no less than 10 in the CES-D-10. This threshold of 10 has been widely used in previous studies [35] and well-validated in depression measurement in Chinese older populations, regardless of their age and dementia status [36, 37]. The internal consistency Cronbach’s alpha value of CES-D-10 is 0.807, which indicates a reasonable level of internal consistency.

### Covariates

To obtain more robust findings, we controlled a wide set of potential confounding factors such as demographics, socioeconomic status, family/social support, health behaviors, and health conditions. The demographic factors included age and sex. Socioeconomic conditions included education, socioeconomic status, rural residence, and geographical regions. Family/social support included marital status and living arrangement. Health behaviors included social and leisure activity index, smoking, alcohol drinking, BMI (as a proxy for unhealthy behaviors), and regular dietary (vegetable/fruit/fish/nut) intake. Health status were measured by self-rated health, cognitive impairment, medical illness, comorbidity, and disability in activities of daily living (ADL). All data were gathered by well-trained research teams through face-to-face home interviews. The sampled older adults were encouraged to answer as many questions as possible asked by the research team member. If the sampled older adult was not able to answer a question or do not the answer, the next-of-kin, the primary caregiver or other informants would be called to serve as proxy to provide the answer [38].

Age was calculated according to self-reported date of birth with validations from various sources such as household registration record (*hukou*), personal identification card, genealogical records, ages of family members, and so forth. Dates were converted into Georgian calendar dates if they were based on Chinese lunar calendar dates. Levels of educational attainment were grouped into three categories according to years of schooling (0, 1–6, and ≥ 7 years). Current residence was dichotomized as “urban residence” or “rural residence”. Given its multidimensionality and complex of socioeconomic status and unavailability of an overall index in the CLHLS, we generated an overall socioeconomic status index by using a principal component analysis (PCA) based on four questions (primary occupation before retirement [white collar vs. others], family economic condition [a Likert scale with five points], retirement earnings, and living expenditure). Following some previous practice [35, 39], a compositional score based on the first component generated from PCA has been suggested to be a qualified measure of socioeconomic status and has been widely employed in previous studies. Marital status was divided as “currently married and living with spouse” or others (widowed, separated, divorced, or never married). Living arrangement was grouped into 3 categories: living with family members or others, living alone, and living in an institution. Social and leisure activity score was calculated by eight types of activities (whether a respondent did gardening, practiced Tai Chi, participated in square dance, raised poultry or pets, reading, playing Mahjong or cards, listening to the radio or watching TV, and participating in community social activities) and we scored each activity 1 for ‘never’, 2 for ‘sometimes’ 3 for ‘almost every day’; The score ranged from 8 to 24 with a higher score indicating more leisure activities, and the low social and leisure activity level was defined by the score less than 14. Smoking status was dichotomized as “non-current smoker or never-smoker” vs. “current smoker”, a similar approach was taken to define alcohol consumption and physical activity. Dietary intake, including vegetables, fruit, fish, and nut, were dichotomized as “regular intake” or “occasional or seldom intake”. The body mass index (BMI) was calculated as weight in kilograms divided by height in meters squared. Cognitive function was tested by using the Chinese version of the 30-point Mini-Mental State Examination (MMSE), and cognitive impairment was defined by an MMSE total score of < 24 [31]. The index of activity of daily living (ADL) was assessed by the Katz index [40], and we defined ADL disability as needing personal assistance in performing one or more of the five essential activities (bathing, transferring, dressing, eating, and toileting) or being incontinent [41]. We ascertained 14 self-reported medical illnesses, including hypertension, diabetes, dyslipidemia, heart disease, stroke, pneumonia (asthma/COPD), cataract or glaucoma, cancer, gastritis, arthritis, cholecystitis, rheumatism, nephritis, and hepatitis; we grouped the medical illness into 3 categories similar to some previous studies [34]: “chronic inflammatory disorders (heart disease, stroke, diabetes, pneumonia, gastritis, arthritis, cholecystitis, rheumatism, nephritis, and hepatitis)”, “other disorders”, and “none”. Comorbidity was defined as having 5 or more medical illnesses. Self-rated health was defined as “excellent or good” or “average or poor”. We considered geographical region on the basis of residential address to account for types of tea production areas [42] as well as differences in regional economic developments and social cultures in China: Northern China (Beijing, Tianjin, Hebei, Shanxi, Shaanxi, Shandong, Liaoning, Jilin, and Heilongjiang provinces), Eastern China (Shanghai, Jiangsu, Zhejiang, and Fujian provinces), Central China (Henan, Hubei, Jiangxi, Anhui, and Hunan provinces), Southwestern China (Guangdong, Guangxi, Chongqing, Sichuan, and Hainan provinces) (Supplementary Figure 1).

### Statistical analyses

The subjects’ characteristics according to categories of type of tea consumption were compared by using analysis of variance or chi-square test, as appropriate. We used multivariate logistic regression analysis to calculate odds ratios (ORs) of depressive symptoms for the type of tea consumption, including green tea, fermented tea, and floral tea, with no habitual tea intake treated as the reference group. The base model (Model 1) included types of tea consumption plus demographic variables; Model 2 further controlled for socioeconomic variables: education, socioeconomic status, rural residence, and geographical regions; Model 3 additionally controlling for psychosocial and behavioral variables: marital status, living arrangement, social and leisure activity index, smoking, alcohol drinking, BMI, regular dietary (vegetable/fruit/fish/nut) intake; Model 4 added health variables in Model 3: self-rated health, cognitive impairment, medical illness, comorbidity, and ADL disability. In detailed analyses examining the dose-effect relation between the intake of green tea or fermented tea or floral tea with depressive symptoms, we classified the frequency of each type of tea consumption into 3 categories: daily (≥ 1 cup/day), occasionally (< 1 cup/day but ≥1 cup/month), and never or rarely (< 1 cup/month or never drink tea), and repeated multiple logistic regressions controlling for all covariates as above.

We conducted subgroup analyses to examine whether the associations between types and frequencies of tea intake and depressive symptoms differed by sex, age (< 80 years old vs. ≥80 years old), residence (urban residence vs. rural residence), and geographical regions (Northern China, Eastern China, Central China, and Southwestern China). We performed several steps of sensitivity analyses for the full model (Model 4) to assess the possible outcomes of the different thresholds used for the CES-D-10. First, we considered varied cut-off thresholds for the CES-D-10, such as 8 and 12, which are more sensitive (cut-off value = 8) or specific (cut-off value = 12) to discriminate the depressive symptoms, and used Model 4 to examine the associations. Second, the participants with severe cognitive impairment with scores of MMSE < 19 were excluded from the present study [43], because of a concerns of their possible recall biases in reporting types and frequencies of tea consumption. Moreover, we excluded older adults who were long bedridden or terminally ill for more robust estimates. We also tested our results by using a full sample after multiple imputations and by adjusting the sampling weight based on the age-sex-residence-specific distribution of the 2015 mini-census of China. STATA version 16.0 (Stata Corp, College Station, TX, USA) was used to perform all analyses. ArcGIS version 10.2 was used to perform map visualization of the geographical distribution of tea drinkers.

## Results

### General characteristics

**Fig. 2:**
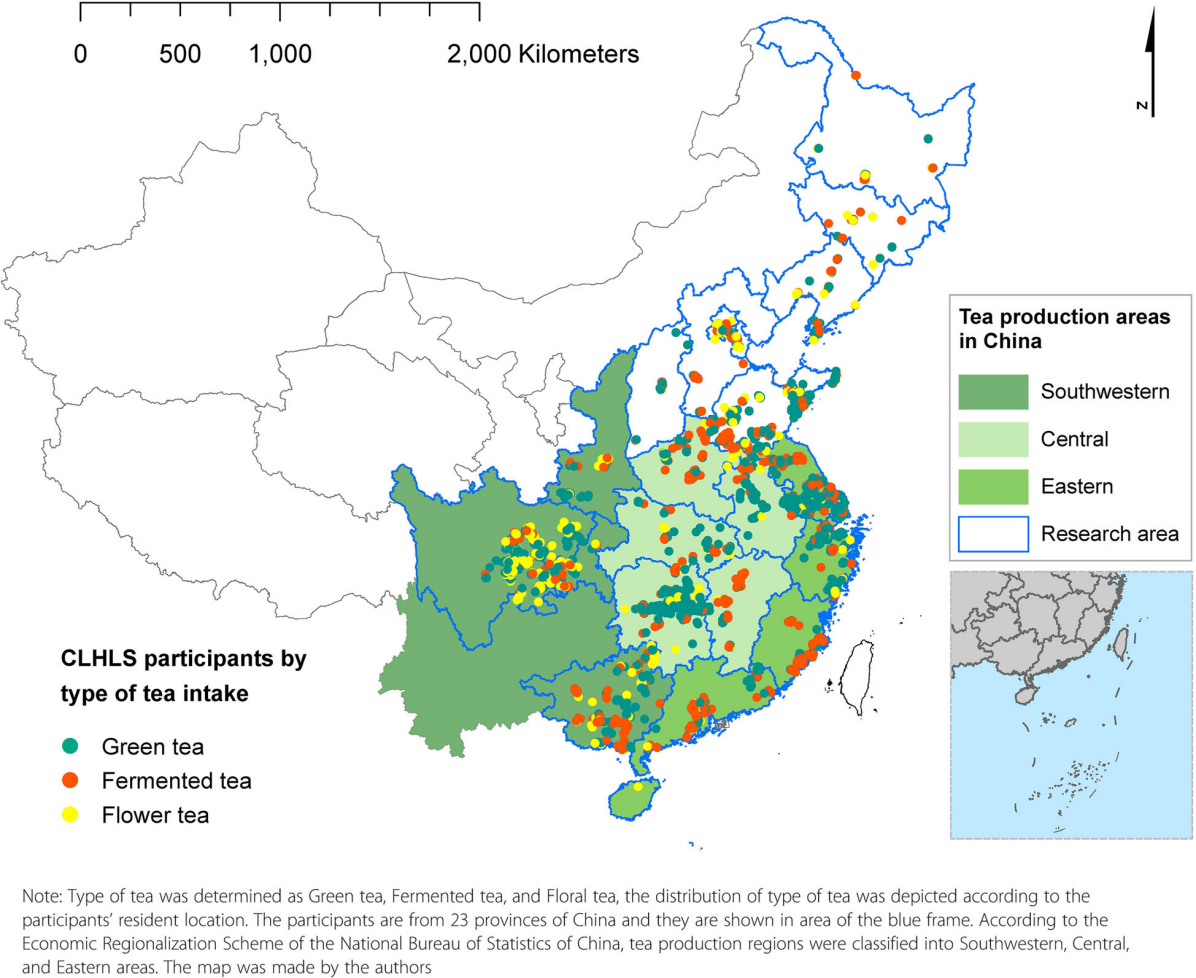
Map of tea production areas in China and distribution of participants in theCLHLS who habitually consumed three main types of tea

The 13,115 study participants with a mean age of 83.7 years (13% aged 100 and above), were evenly distributed across the whole of China in four geographical regions and diversified by socioeconomic, lifestyle, and health-related characteristics (Table 1). Among them, the mean CES-D-10 score was 10.1 (SD: 4.7); 56.6% of the study participants showed a CES-D-10 score of ≥10 indicating depression. Overall, 70.3% of the study participants never or rarely consumed tea, 15.0% consumed green tea, 8.8% consumed fermented tea, and 5.9% consumed floral tea. They were widely distributed geographically, with fermented tea consumption relatively more concentrated in the Eastern tea production Region, and green tea in the Central Region (Fig. 2). Compared to non-drinkers, tea drinkers as a whole in the 2018 CLHLS sample were significantly younger, predominantly men, more likely to be married, urban rather than rural dwellers, more active in social and leisure activity, and had a higher socioeconomic status. However, tea drinkers were more likely to be smokers and alcohol drinkers as well. On the other hand, them were more likely to report regular intake of vegetables, fruits, nuts, and fish. Their prevalence rates of reported chronic diseases, including chronic inflammatory diseases and comorbidity, were higher.

**Table 1:** Main characteristics of the whole study population and its subgroups by type of tea consumption^a^

Variable/subgroups	Total sample	No tea	Green tea	Fermented tea	Floral tea	*P* value^b^
Total sample, n	13,115	9220	1970	1156	769	
%	100.0	70.3	15.0	8.8	5.9	
CES-D-10						
Total score	10.1 ± 4.7	10.4 ± 4.6	9.6 ± 4.8	9.5 ± 4.8	9.1 ± 4.8	< 0.001
≥ 10	7419 (56.6)	5454 (59.2)	1010 (51.3)	590 (51.0)	365 (47.5)	< 0.001
Age, years	83.7 ± 11.2	84.5 ± 11.3	81.4 ± 10.7	81.9 ± 11.0	82.6 ± 11.1	< 0.001
Age group, years						< 0.001
65–79	5121 (39.1)	3319 (36.0)	924 (46.9)	537 (46.5)	341 (44.3)	
80–99	6301 (48.0)	4577 (49.6)	868 (44.1)	514 (44.5)	342 (44.5)	
≥ 100	1693 (12.9)	1324 (14.4)	178 (9.0)	105 (9.1)	86 (11.2)	
Women (%)	7102 (54.2)	5574 (60.5)	689 (35.0)	521 (45.1)	451 (41.4)	< 0.001
Education level, years						< 0.001
0	7003 (53.4)	5487 (59.5)	686 (34.8)	511 (44.2)	319 (41.5)	
1–6	2643 (20.2)	1798 (19.5)	421 (21.4)	238 (20.6)	186 (24.2)	
7+	3469 (26.5)	1935 (21.0)	863 (43.8)	407 (35.2)	364 (34.3)	
Rural residence, %	5782 (44.1)	4335 (47.0)	726 (36.9)	446 (38.6)	275 (35.8)	< 0.001
Socioeconomic status ^c^	−0.17 ± 1.00	0.08 ± 0.87	−0.31 ± 1.35	− 0.16 ± 1.01	−0.24 ± 1.10	< 0.001
Married and living with a spouse, %	5676 (43.3)	3604 (39.1)	1092 (55.4)	597 (51.6)	386 (49.8)	< 0.001
Living arrangement %						< 0.001
With household members	10,504 (80.1)	7287 (79.0)	1647 (83.6)	926 (80.1)	644 (83.8)	
Alone	2178 (16.6)	1620 (17.6)	268 (13.6)	191 (16.5)	99 (12.9)	
Institution	433 (3.3)	313 (3.4)	55 (2.8)	39 (3.4)	26 (3.4)	
Social and leisure activity index	3.90 ± 2.76	3.53 ± 2.63	4.91 ± 2.90	4.64 ± 2.82	4.55 ± 2.86	< 0.001
Self-rated health excellent or good, %	6317 (48.2)	4307 (46.7)	1019 (51.7)	596 (51.6)	395 (51.4)	< 0.001
Current or former Smoker, %	4045 (30.8)	2312 (25.1)	922 (46.8)	470 (40.7)	341 (44.3)	< 0.001
Current alcohol drinker, %	1985 (15.1)	1124 (12.2)	482 (24.5)	944 (18.3)	167 (21.7)	< 0.001
BMI	22.4 ± 4.2	22.2 ± 4.2	22.8 ± 3.9	22.8 ± 4.2	23.4 ± 4.3	0.108
Regular vegetable consumption, %	11,827 (90.2)	8245 (89.4)	1819 (92.3)	1069 (92.5)	694 (90.2)	< 0.001
Regular fruit consumption, %	6104 (46.5)	4041 (43.8)	1038 (52.7)	620 (53.6)	405 (52.7)	< 0.001
Regular fish intake, %	6301 (48.6)	4064 (44.6)	1197 (61.5)	665 (57.8)	375 (49.4)	< 0.001
Regular nut intake, %	2539 (19.6)	1510 (16.6)	521 (26.8)	252 (21.9)	256 (33.8)	< 0.001
Regularly physical activity, %	9651 (73.6)	6945 (75.3)	1336 (67.8)	842 (72.8)	528 (68.7)	< 0.001
Cognitive impairment, %	3300 (25.2)	2556 (27.7)	338 (17.2)	269 (23.3)	137 (17.8)	< 0.001
ADL functional disability, %	2974 (22.7)	2214 (24.0)	346 (17.6)	210 (18.2)	204 (26.5)	
Medical illness, %						< 0.001
Chronic inflammatory disorders ^d^	5714 (43.6)	3900 (42.3)	915 (46.5)	528 (45.7)	371 (48.2)	
Other disorders	4607 (35.1)	3295 (35.7)	650 (33.0)	408 (35.3)	254 (33.0)	
None	2794 (21.3)	2025 (22.0)	405 (20.6)	220 (19.0)	144 (18.7)	
Comorbidity ^e^, %						< 0.001
Yes	606 (4.6)	376 (4.1)	124 (6.3)	49 (4.2)	57 (7.4)	
No	12,509 (95.4)	8844 (95.9)	1846 (93.7)	1107 (95.8)	712 (92.6)	
Geographical region ^f^, %						< 0.001
Northern China	3157	1969 (62.4)	506 (16.0)	301 (9.5)	381 (12.1)	
Eastern China	2842	1933 (68.0)	607 (21.4)	258 (9.1)	44 (1.5)	
Central China	2967	2307 (77.8)	435 (14.7)	173 (5.8)	52 (1.8)	
Southwestern China	4149	3011 (72.6)	422 (10.2)	424 (10.2)	292 (7.0)	

^a^ The percentages in the parentheses are unweighted and refer to those within each type of tea. *CES-D-10* 10-item of Center for Epidemiological Studies Depression Scale, *BMI* body mass index, *ADL* activity of daily living

^b^ Based on chi-square test (n, %) or ANOVA (mean ± SD)

^c^ Based the first factor score of PCA from four variables (occupation, economic conditions, family income and expense)

^d^ Chronic inflammatory disorder was determined as having at least one condition of cardiovascular diseases/stroke, diabetes, asthma/COPD, arthritis, cholecystitis, nephritis, hepatitis, and gastric or duodenal ulcer

^e^ Comorbidity was determined as comorbid 5 or more in 14 medical illnesses consisting of hypertension, diabetes, dyslipidemia, heart disease, stroke, pneumonia (asthma/COPD), cataract or glaucoma, rheumatism, cancer, arthritis, cholecystitis, nephritis, hepatitis, and gastric or duodenal ulcer

^f^ Geographical regions were considered on the basis of residential address to account for tea consumption habits and dietary differences in China: Northern China (Beijing, Tianjin, Hebei, Shanxi, Shaanxi, Shandong, Liaoning, Jilin, Heilongjiang), Eastern China (Shanghai, Jiangsu, Zhejiang, Fujian), Central China (Henan, Hubei, Jiangxi, Anhui, Hunan), and Southwestern China (Guangdong, Guangxi, Chongqing, Sichuan, Hainan)

### Associations of type and frequency of tea intake with depressive symptoms

Tea drinkers showed lower odds of having depressive symptoms in model controlling for demographic and socioeconomic variables: green tea, OR = 0.72 (95% CI: 0.65–0.80); fermented tea, OR = 0.79 (95% CI: 0.70–0.89); floral tea, OR = 0.62 (95% CI: 0.53–0.72) (Table 2). The magnitude of association was reduced after further controlling for psychological, lifestyle, behavioral, and health variables, but the final model controlling for all confounding risk factors showed that tea consumption remained associated with 15% (green tea) to 30% (floral tea) lower odds of having depressive symptoms. Stratified analyses showed some heterogeneity of associations by tea type, sex, age group, and geographical region: green tea in men (OR = 0.79) versus women (OR = 0.93), floral tea in men (OR = 0.77) versus women (OR = 0.61), fermented tea in the young-old group (OR = 0.81) versus the oldest-old group (OR = 0.96), green tea in Southwestern region (OR = 0.60) versus other regions (OR from 0.83 to 0.90), fermented tea in Central and Southwestern regions (OR = 0.70 and 0.76) versus Northern and Eastern regions (OR = 1.09 and 1.11), and floral tea in Eastern China (OR = 0.48) versus other regions (OR from 0.70 to 0.88).

**Table 2:** Odds ratios of depressive symptoms by type of tea consumption among whole sample and subpopulations

	Green tea vs. No tea	Fermented tea vs. No tea	Floral tea vs. No tea
**Whole sample**^**a**^			
Model 1	0.53 (0.48–0.58) ^*^	0.63 (0.56–0.70) ^*^	0.49 (0.43–0.57) ^*^
Model 2	0.72 (0.65–0.80) ^*^	0.79 (0.70–0.89) ^*^	0.62 (0.53–0.72) ^*^
Model 3	0.80 (0.72–0.89) ^*^	0.84 (0.74–0.95) ^*^	0.69 (0.60–0.81) ^*^
Model 4	0.85 (0.76–0.95) ^*^	0.87 (0.76–0.99) ^*^	0.70 (0.59–0.82) ^*^
**Subpopulations based on model 4**			
By Sex			
Men	0.79 (0.69–0.92) ^*^	0.85 (0.71–1.03)	0.77 (0.62–0.95) ^*^
Women	0.93 (0.79–1.10)	0.89 (0.74–1.08)	0.61 (0.48–0.78) ^*^
By Age group			
Age < 80 years	0.87 (0.75–1.00)	0.81 (0.68–0.97) ^*^	0.70 (0.56–0.87) ^*^
Age ≥ 80 years	0.82 (0.69–0.97) ^*^	0.96 (0.79–1.18)	0.69 (0.54–0.88) ^*^
By urban-rural residence			
Urban residency	0.82 (0.73–0.94) ^*^	0.90 (0.76–1.07)	0.71 (0.58–0.88) ^*^
Rural residency	0.91 (0.76–1.08)	0.84 (0.68–1.04)	0.68 (0.52–0.89) ^*^
By geographic region			
Northern China	0.84 (0.66–1.06)	1.09 (0.83–1.44)	0.74 (0.57–0.96) ^*^
Eastern China	0.90 (0.74–1.11)	1.11 (0.85–1.46)	0.48 (0.25–0.94) ^*^
Central China	0.83 (0.67–1.04)	0.76 (0.55–1.05)	0.70 (0.39–1.28)
Southwestern China	0.60 (0.47–0.76) ^*^	0.70 (0.56–0.87) ^*^	0.88 (0.68–1.03)

^a^ Model 1 included types of tea consumption as the sole variable; Model 2 controlling for demographic and socioeconomic variables: age (continuous), sex, education, socioeconomic status, rural residence and geographical regions; Model 3 additionally controlling for psychosocial and behavioral variables: marital status, living arrangement, social and leisure activity index, smoking, alcohol drinking, BMI, regular dietary (vegetable/fruit/fish/nut) intake; Model 4 additionally for health variables: self-rated health, cognitive impairment, and medical illness, comorbidity, and ADL disability

^*^ *P* < 0.05

**Fig. 3:**
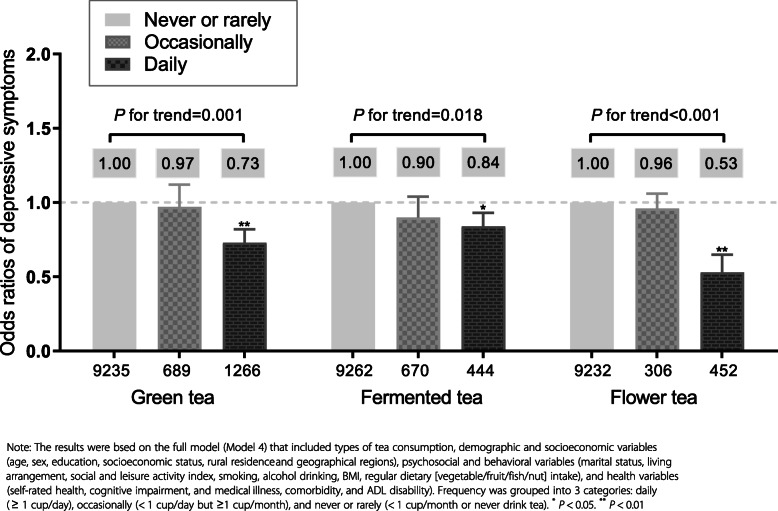
Types and frequencies of habitual tea consumption and depressive symptoms

The frequencies of intaking green, fermented, and floral tea all showed linear associations respectively with depressive symptoms, controlling for major potential confounding factors (Fig. 3). The associations were more marked for green tea (*P* = 0.001 for a linear trend) and floral tea (*P* = 0.001 for a linear trend). Daily drinking of one or more cups of tea of all three types of tea was significantly associated with 16% (fermented tea), 27% (green tea), and 47% (floral tea) lower odds of presence of depressive symptoms. Those associations were generally consistent in the subgroup analyses by sex and residence, while the associations of green tea and floral tea intake with depressive symptoms were more pronounced in the oldest-old group (≥ 80 years) compared to the young-old group (65–79 years). In the oldest-old subsample with never or rarely tea intake group as the reference, the ORs of depressive symptoms for daily green tea drinkers and daily floral tea drinkers were 0.72 (95% CI: 0.60, 0.86) and 0.47 (95% CI: 0.35, 0.64), respectively; yet the ORs were 0.82 (95% CI: 0.67, 1.01) and 0.60 (95% CI: 0.42, 0.86) for daily green tea drinkers and daily floral tea drinkers in the young-old groups. We also observed a geographical variation in the associations in green tea and fermented tea consumers, while the association seems more homogeneous in floral tea drinkers (Supplemental Table 2).

### Sensitivity analyses

In sensitivity analysis using varied cut-off values of the CES-D-10 such as 8 and 12, we repeated the analysis for the full model (Model 4). The dose-effect relationship of frequencies of each type of tea intake with depressive symptom was only mildly altered in analyses using both cut-offs (Supplementary Figure 2). After excluding participants who were likely to have severe cognitive impairment (defined by a score of MMSE less than 19, *n* = 1432), the estimates or the significance levels of the observed associations were not altered in three types of tea drinkers. Moreover, we removed the participants who were long bedridden or terminally ill (*n* = 261), restricting the sample to non-bedridden and the results were identical to those we presented in the main text (Supplementary Figure 3). We also tested our result using a full sample after multiple imputations and further adjusting the sampling weight (Supplementary Figure 4). Those sensitivity analyses were all reasonably consistent with the final model in the main text.

## Discussion

In this large population-based study, Chinese older adults who regularly consumed all types of tea (green, fermented, and floral) were less likely to show depressive symptoms, measured by the CES-D-10. Daily consumption of one or more cups of green, fermented, or floral tea was associated with up to 50% lowered odds of having depressive symptoms. These findings are in line with previous observations of an inverse association of tea consumption with the risk of depression [22–27].

There are several noteworthy aspects of the association observed in this study that are not present in previous studies. One is that several types of tea consumption were investigated simultaneously in the study. Most prior studies mainly investigated only one type of tea alone [21, 24]; or unspecified tea type [18, 22, 23, 26]. In two studies, green and black (fermented) tea were both investigated [25, 26]. One study in rural North China reported lower odds of depressive symptoms for green and black tea [26], similar to our findings, whereas another study in Eastern China reported an inverse association of black tea and depression, but not for green tea [25]. Our study reveals additionally an inverse association of floral tea with depression, which was also observed with marginal significance in the study in North China [26].

Tea is one of the most widely consumed beverages in the world, both in the traditional ways of drinking and as a constituent of ready-to-drink beverages. In addition to green tea and black tea, some types of tea are becoming increasingly sold in the Western world, such as white tea, yellow tea, dark tea, matcha, and floral tea [28]. The main chemical compounds were varied between different types of teas due to varied processes, which can be summarized as withering, fixation, rolling, fermentation, and drying steps. (−)-epigallocatechin gallate, trans-catechins, caffeine, and theanine are the main compounds of green, white, and oolong teas, which account for about 20 to 30% of the dry weight. In black tea, trans-catechins are scarcely detected, but gallic acid, caffeine, and theaflavin are the major compounds [44]. In our study, we observed a lower prevalence of depressive symptoms in green tea drinkers than those who were fermented tea drinkers. The more pronounced antidepressant effect of green teas can be partly due to the antioxidant and anti-inflammatory components such as catechins or EGCG. In general, green tea has been found to be superior to fermented tea in terms of antioxidant activity owing to the higher content of (−)-epigallocatechin gallate [32]. Notably, consuming at least one cup of floral tea per day is significantly associated with lower odds of having depressive symptoms, compared to those non-drinkers. Studies identified effective constituents of floral teas, such as Okanin, which can exert a neuroprotective effect through inhibition of the TLR4/NF-κB signaling pathways [45]. Clinical trial data indicate that consuming chamomile tea, one of the most commonly consumed floral tea, can attenuate depression state in depressed patients with type 2 diabetes [46] and in postpartum women [47]. Similar antidepressant and sedative effect of jasmine tea, especially for its odor such as (*R*)-(−)-linalool, was also reported [48, 49].

The association of tea consumption with depression is observed to be highly heterogeneous among studies and populations across the world, and is true within China, as evidenced by the data in this study. The heterogeneity may be partly explained by the local popularity of different types of tea used for consumption and methods of infusion and preparation in production [27, 50]. In China, tea of different types is traditionally consumed on its own without or rarely with milk and remains so today, whereas tea, mostly of mixed blends of black fermented tea, is popularly consumed with milk in the West [51]. Some evidence suggests that the addition of milk may reduce the antioxidant activity of tea, due to the interaction between tea polyphenols and milk proteins, such as between catechins and caseins, among other factors [52, 53]. However, more research is warranted to shed light on this.

We also observed some variations of the associations by age and sex. The association of green tea intake and depressive symptoms seems more marked in older men, while the association of floral tea and depressive symptoms was more pronounced in older women. The associations of green tea and floral tea intakes with depressive symptoms were generally consistent in the young-old group (Age < 80 years) and in the oldest-old group (Age ≥ 80 years), while the association was more marked in the young-old group regarding the fermented tea intaking. As we did not find significant interaction between the groups by sex and age, we decided to interpret our results in a more cautious way. Further studies are warranted to examine the associations of varied types of tea intake and depressive symptoms regarding the sex differences and heterogeneity by age.

Our study had several strengths. To our knowledge, this is the first study that has investigated the association of different types and frequencies of tea intake with depressive symptoms among a nationally representative sample of older adults in China. We also did subgroup investigations, especially among the oldest-old participants and participants with varied geographical regions. Besides, we considered a wide range of covariates that allowed us to include and adjust for major potential confounders that were measured in the study population. Moreover, our study had a large sample size, which allowed us to test the associations between varied types and frequencies of tea consumption and various grades of depressive symptoms (using different cut-offs of CES-D-10 from 8, 10, and 12).

Several methodologic limitations should be considered in the interpretation of our results. First, our study had a cross-sectional design, which prevented us from firmly establishing a causal relationship between the consumption of each type of tea and depressive symptoms. Second, different from some previous studies using more detailed data on tea consumption, the present study used very basic patterns of frequency of tea consumption among older Chinese, which may bias the findings. Studies with accurate amount of tea consumption would be more informative. Third, the estimated inverse odds ratio of the association between tea consumption and depressive symptoms were substantially attenuated by the additional inclusion of multiple covariates in the model, suggesting that the effect of tea consumption on depressive symptoms was explained in large parts by its association with socioeconomic, psychosocial, lifestyle behavioral, and health-related factors. For example, healthier and socially active individuals with higher socioeconomic status tended to have more opportunities to consume varied types of teas [54]. Among the Chinese, tea is often consumed as a social or leisure activity [55], and such a social or leisure activity itself, as well as the process of preparing and drinking tea, may contribute to maintaining better mental health [56, 57]. It is also likely that such attenuation was due to the presence of associations between depression symptoms and socioeconomics, psychosocial, and other behavioral factors as evidenced by previous research [58]. In addition, tea consumption has also been shown to be associated with lower risks of cardio-metabolic risks [58, 59], which in turn have also been demonstrated to be associated with depression [60]. It is also likely that such attenuation was due to the presence of associations between depression symptoms and socioeconomics, psychosocial, and other behavioral factors as evidenced by previous research. Furthermore, inflammation may be a common underlying factor in this relationship, as it is associated with many chronic diseases and depression [61]. Although we were able to control for chronic inflammatory disorders and many other potential confounders, and the findings were generally robust to adjustments, we may not be able to fully exclude the possibility of residual confounding by unmeasured factors. Finally, although the CES-D-10 is well validated in assessing depression in Chinese older populations [36], there was lack of clinical assessment of depression in the community-based survey [62], hence we were not able to diagnose the presence of clinical depression or the subtype of depression. More interventional studies and clinical trials among general healthy populations as well as clinically depressed patients are warranted to assess the generalizability of the present findings.

## Conclusions

In conclusion, this large Chinese population-based study demonstrated that a higher consumption of tea, including green, fermented, floral tea, was inversely associated with the prevalence of depressive symptoms, while the association was particularly pronounced among floral tea drinkers. These findings suggest that the consumption of various types of tea may be potentially beneficial for the prevention of depressive symptoms. Prospective studies or randomized trials are required to clarify the causality, taking into account the types of tea.

## Supplementary Information

**Additional file 1: Supplementary Table 1**. Types and frequencies of consumption of teas in the study population. **Supplementary Table 2**. Subgroup analyses on levels and types of tea consumption and depressive symptoms. **Supplementary Figure 1**. Map of tea production areas in China and distribution of participants of CLHLS by type of tea intake . **Supplementary Figure 2**. Sensitivity analysis of type and frequency of tea intake with depressive symptoms by (a) using different cut-off value of CES-D-10 = 12 and (b) using different cut-off value of CES-D-10 = 8 as the definition of depressive symptoms. Main model included types of tea consumption as the sole variable and controlling for demographic and socioeconomic variables (age, gender, education, socioeconomic status, rural residence and geographical regions), psychosocial and behavioral variables (marital status, living arrangement, social and leisure activity index, smoking, alcohol drinking, BMI, regular dietary [vegetable/fruit/fish/nut] intake), and health variables (self-rated health, cognitive impairment, and medical illness, comorbidity, and ADL disability). **Supplementary Figure 3**. Sensitivity analysis of type and frequency of tea intake with depressive symptoms by (a) removing participants with severe cognitive impairment (MMSE< 19; *n* = 1432) and (b) removing participants who were bedridden or terminally ill (*n* = 261). Main model included types of tea consumption as the sole variable and controlling for demographic and socioeconomic variables (age, gender, education, socioeconomic status, rural residence and geographical regions), psychosocial and behavioral variables (marital status, living arrangement, social and leisure activity index, smoking, alcohol drinking, BMI, regular dietary [vegetable/fruit/fish/nut] intake), and health variables (self-rated health, cognitive impairment, and medical illness, comorbidity, and ADL disability). **Supplementary Figure 4**. Sensitivity analysis of type and frequency of tea intake with depressive symptoms by (a) using full sample after multiple imputation (*n* = 13,825) and (b) by adjusting sampling weight based on age-sex-residence-specific distribution of 2015 mini-census of China. Main model included types of tea consumption as the sole variable and controlling for demographic and socioeconomic variables (age, gender, education, socioeconomic status, rural residence and geographical regions), psychosocial and behavioral variables (marital status, living arrangement, social and leisure activity index, smoking, alcohol drinking, BMI, regular dietary [vegetable/fruit/fish/nut] intake), and health variables (self-rated health, cognitive impairment, and medical illness, comorbidity, and ADL disability).

## Data Availability

The data that support the findings of this study are available in https://opendata.pku.edu.cn/dataverse/CHADS, per the reasonable request.

## Abbreviations

ADL: Activity of daily living
CLHLS: Chinese Longitudinal Healthy Longevity Survey
CED-S-10: 10-item Center for Epidemiologic Studies Depression Scale
ECCG: Epigallocatechin gallate
MMSE: Mini-Mental State Examination
