# Supplementary material for: Type of tea consumption and depressive symptoms in Chinese older adults
Source: BMC Geriatr. 2021 May 24;21:331. doi: 10.1186/s12877-021-02203-z (PMC8142291; doi:10.1186/s12877-021-02203-z)
Supplement: Supplementary file 1 — Additional file 1: Supplementary Table 1. Types and frequencies of consumption of teas in the study population. Supplementary Table 2. Subgroup analyses on levels and types of tea consumption and depressive symptoms. Supplementary Figure 1. Map of tea production areas in China and distribution of participants of CLHLS by type of tea intake . Supplementary Figure 2. Sensitivity analysis of type and frequency of tea intake with depressive symptoms by (a) using different cut-off value of CES-D-10 = 12 and (b) using different cut-off value of CES-D-10 = 8 as the definition of depressive symptoms. Main model included types of tea consumption as the sole variable and controlling for demographic and socioeconomic variables (age, gender, education, socioeconomic status, rural residence and geographical regions), psychosocial and behavioral variables (marital status, living arrangement, social and leisure activity index, smoking, alcohol drinking, BMI, regular dietary [vegetable/fruit/fish/nut] intake), and health variables (self-rated health, cognitive impairment, and medical illness, comorbidity, and ADL disability). Supplementary Figure 3. Sensitivity analysis of type and frequency of tea intake with depressive symptoms by (a) removing participants with severe cognitive impairment (MMSE< 19; n = 1432) and (b) removing participants who were bedridden or terminally ill (n = 261). Main model included types of tea consumption as the sole variable and controlling for demographic and socioeconomic variables (age, gender, education, socioeconomic status, rural residence and geographical regions), psychosocial and behavioral variables (marital status, living arrangement, social and leisure activity index, smoking, alcohol drinking, BMI, regular dietary [vegetable/fruit/fish/nut] intake), and health variables (self-rated health, cognitive impairment, and medical illness, comorbidity, and ADL disability). Supplementary Figure 4. Sensitivity analysis of type and frequency of tea int [file 12877_2021_2203_MOESM1_ESM.pdf]

Supplementary Contents

Type of tea consumption and depressive symptom in older women and men

Yao Yao, Huashuai Chen, Lele Chen, Sang-Yhun Ju, Huazhen Yang, Yi Zeng, Danan Gu, and Tze Pin NG

Supplementary Table 1……………………………………………………………………………………………… **Page 2**
Types and frequencies of consumption of teas in the study population.

Supplementary Table 2……………………………………………………………………………………………… **Page 3**
Subgroup analyses on levels and types of tea consumption and depressive symptoms

Supplementary Figure 1…………………………………………………………………………………………….. **Page 5**
Map of tea production areas in China and distribution of participants of CLHLS by type of tea intake

Supplementary Figure 2…………………………………………………………………………………………….. **Page 6**
Sensitivity analysis of type and frequency of tea intake with depressive symptoms by (a) using different cut-off value of CES-D-10=12 and (b) using different cut-off value of CES-D-10=8 as the definition of depressive symptoms.

Supplementary Figure 3…………………………………………………………………………………………….. **Page 7**
Sensitivity analysis of type and frequency of tea intake with depressive symptoms by (a) removing participants with severe cognitive impairment (MMSE<19; n=1,432) and (b) removing participants who were bedridden or terminally ill (n=261).

Supplementary Figure 4…………………………………………………………………………………………….. **Page 8**
Sensitivity analysis of type and frequency of tea intake with depressive symptoms by (a) using full sample after multiple imputation (n=13,825) and (b) by adjusting sampling weight based on age-sex-residence-specific distribution of 2015 mini-census of China.

Supplementary Contents

Supplementary Table 1

Types and frequencies of consumption of teas in the study population.

Types of tea consumption			Detailed classifications
**Green tea**			Bi Luo Chun, Lung Chen tea (Dragon Well tea), Anhua pine needles, Baohong tea, Xinyang Maojian, Emei bamboo leaf green, Liuan melon slices, Duyun Maojian, En Shi Yulou, Gaoqiao Yinfeng, Guzhu purple bamboo shoots, Guiping Xishan tea, Huangshan Maofeng, Huiming tea, Jietan tea, Trail Mountain tea, Jingting green snow, Old Bamboo generous, Mount Lu Yunwu tea, Mei tea, Mengding tea, Nan'an Shiting green, Nanjing Rain Flower tea, Pingshui Zhu tea, Qinba Wuhao, Quan Ganghun Bai, Rizhu snow bud, Taiping Monkey Kui, Tianshan green bud, Tianzun Gong bud, Wuxi Hao tea, Wu Zi Xianhao, Wuyuan Minmei, Wuzhou Ju Yan, Xiuzhou Bi Feng, Cactus tea, Huoning Songluo, Chungxi fire green, Yunfeng and coiling Hao, Zi-Yang Maojian, etc.
**Fermented tea**	Low	White tea	White peony, Gongmei, Shoumei, New craft white tea, Silver needle Pekoe, etc.
	↓	Yellow tea	Beigang Mao Jian, Guangdong Da Ye Qing, Seahorse Palace tea, Huoshan Snow Bud, Junshan Silver Needle, Luyuan Mao Jian, Meng Ding Yellow Bud, Anhui West Yellow Tea, Weishan Baimaojian, Wenzhou Huangtang, etc.
	degree of fermentation	Oolong tea	Tie Guanyin, Anxi color tea, Ba Jiao Ting Long Shu tea, Northern Fujian Narcissus, White Hair Monkey, Phoenix Narcissus, Golden Cinnamon, Wuyi Cinnamon, Taiwan Oolong, Yongchun Buddha Hand, Wuyi Rock Tea, etc.
	↓	Black tea	Chuan red tea, Keemun red tea, small seed red tea, Dian red tea, red tea, Lake red tea, Min red tea, Ning red tea, Yi red tea, Yue red tea, etc.
	↓	Compressed tea	Cake tea, Square bun tea, Porcupine brick tea, Solid tea, Black brick tea, Tight tea, Kang brick and golden tip tea, Rice brick tea, Pu-tuo square tea, Cyan brick tea, Tuo tea, Xiang-tip tea, Round tea, Bamboo fragrant tea, etc.
	High	Dark tea	Hunan black tea, Old cyan tea, Six Fort loose tea, Pu-erh tea, etc.
**Flower tea**			White orchid tea, May flower tea, Chamomile tea, Chrysanthemum tea, Lavender tea, Honeysuckle tea, Rose tea, Jasmine tea, etc.

Frequency of tea consumption	Green tea	Fermented tea	Flower tea
Never or rarely: < 1 cup/month or never drink tea	9235	9262	9232
Occasionally: < 1 cup/day but ≥ 1 cup/month	689	670	306
Daily: ≥ 1 cup/day	1266	444	452

Supplementary Contents

Supplementary Table 2

Subgroup analyses on levels and types of tea consumption and depressive symptoms

	Green tea			Fermented tea			Flower tea		
	Never or rarely	Occasionally	Daily	Never or rarely	Occasionally	Daily	Never or rarely	Occasionally	Daily
Whole sample	9235	689	1266	9262	670	444	9232	306	452
OR (95% CI)[1]									
Model 1	1.00	0.70 (0.60-0.81)	0.44 (0.39-0.50)	1.00	0.73 (0.64-0.85)	0.47 (0.39-0.57)	1.00	0.74 (0.62-0.89)	0.34 (0.28-0.41)
Model 2	1.00	0.89 (0.76-1.03)	0.64 (0.56-0.72)	1.00	0.84 (0.73-0.98)	0.67 (0.56-0.82)	1.00	0.87 (0.70-1.08)	0.45 (0.36-0.55)
Model 3	1.00	0.95 (0.82-1.10)	0.70 (0.61-0.79)	1.00	0.88 (0.76-1.03)	0.75 (0.61-0.91)	1.00	0.94 (0.75-1.15)	0.49 (0.40-0.61)
Model 4	1.00	0.97 (0.80-1.15)	0.73 (0.66-0.87)	1.00	0.90 (0.76-1.04)	0.84 (0.75-0.96)	1.00	0.96 (0.76-1.20)	0.53 (0.43-0.68)
Subgroup Analyses based on Model 4									
Males									
OR (95% CI)	1.00	0.99 (0.79-1.24)	0.71 (0.59-0.84)	1.00	0.86 (0.66-1.13)	0.84 (0.65-1.07)	1.00	1.25 (0.91-1.74)	0.54 (0.40-0.72)
Females									
OR (95% CI)	1.00	1.01 (0.79-1.28)	0.86 (0.69-1.07)	1.00	0.90 (0.73-1.12)	0.85 (0.70-1.00)	1.00	0.71 (0.51-0.97)	0.50 (0.35-0.72)
Age < 80 years									
OR (95% CI)	1.00	1.04 (0.80-1.35)	0.82 (0.67-1.01)	1.00	0.86 (0.63-1.18)	0.74 (0.56-0.97)	1.00	0.97 (0.66-1.42)	0.60 (0.42-0.86)
Age ≥ 80 years									
OR (95% CI)	1.00	0.98 (0.80-1.21)	0.72 (0.60-0.86)	1.00	0.97 (0.79-1.19)	0.85 (0.63-1.11)	1.00	0.94 (0.70-1.26)	0.47 (0.35-0.64)
Urban residency									
OR (95% CI)	1.00	0.96 (0.78-1.18)	0.73 (0.62-0.87)	1.00	0.97 (0.79-1.19)	0.78 (0.59-1.03)	1.00	1.07 (0.79-1.45)	0.52 (0.39-0.68)
Rural residency									
OR (95% CI)	1.00	1.09 (0.84-1.43)	0.81 (0.66-1.01)	1.00	0.76 (0.59-1.00)	0.97 (0.71-1.35)	1.00	0.82 (0.57-1.17)	0.54 (0.37-0.80)
Northern China									
OR (95% CI)	1.00	1.05 (0.76-1.46)	0.75 (0.56-1.01)	1.00	1.35 (0.89-1.72)	0.61 (0.37-1.00)	1.00	1.02 (0.71-1.47)	0.56 (0.40-0.79)
Eastern China									
OR (95% CI)	1.00	1.25 (0.91-1.62)	0.85 (0.67-1.07)	1.00	0.91 (0.64-1.29)	1.40 (0.95-1.86)	1.00	0.65 (0.31-1.55)	0.43 (0.18-1.05)
Central China									

OR (95% CI)	1.00	0.88 (0.62-1.25)	0.85 (0.65-1.11)	1.00	0.74 (0.50-1.08)	0.83 (0.43-1.60)	1.00	0.72 (0.30-1.68)	0.63 (0.29-1.38)
Southwestern China									
OR (95% CI)	1.00	0.81 (0.57-1.15)	0.48 (0.35-0.65)	1.00	0.71 (0.54-0.93)	0.68(0.48-1.98)	1.00	1.08 (0.83-1.37)	0.63 (0.44-0.91)

[1]OR, odds ratio; 95% CI, 95% confidential interval. Model 1 included types of tea consumption as the sole variable; Model 2 controlling for demographic and socioeconomic variables: age (continuous), gender, education, socioeconomic status, rural residence and geographical regions; Model 3 additionally controlling for psychosocial and behavioral variables: marital status, living arrangement, social and leisure activity index, smoking, alcohol drinking, BMI, regular dietary (vegetable/fruit/fish/nut) intake; Model 4 additionally for health variables: self-rated health, cognitive impairment, and medical illness, comorbidity, and ADL disability.

Supplementary Contents

Supplementary Figure 1

Map of tea production areas in China and distribution of participants of CLHLS by type of tea intake.

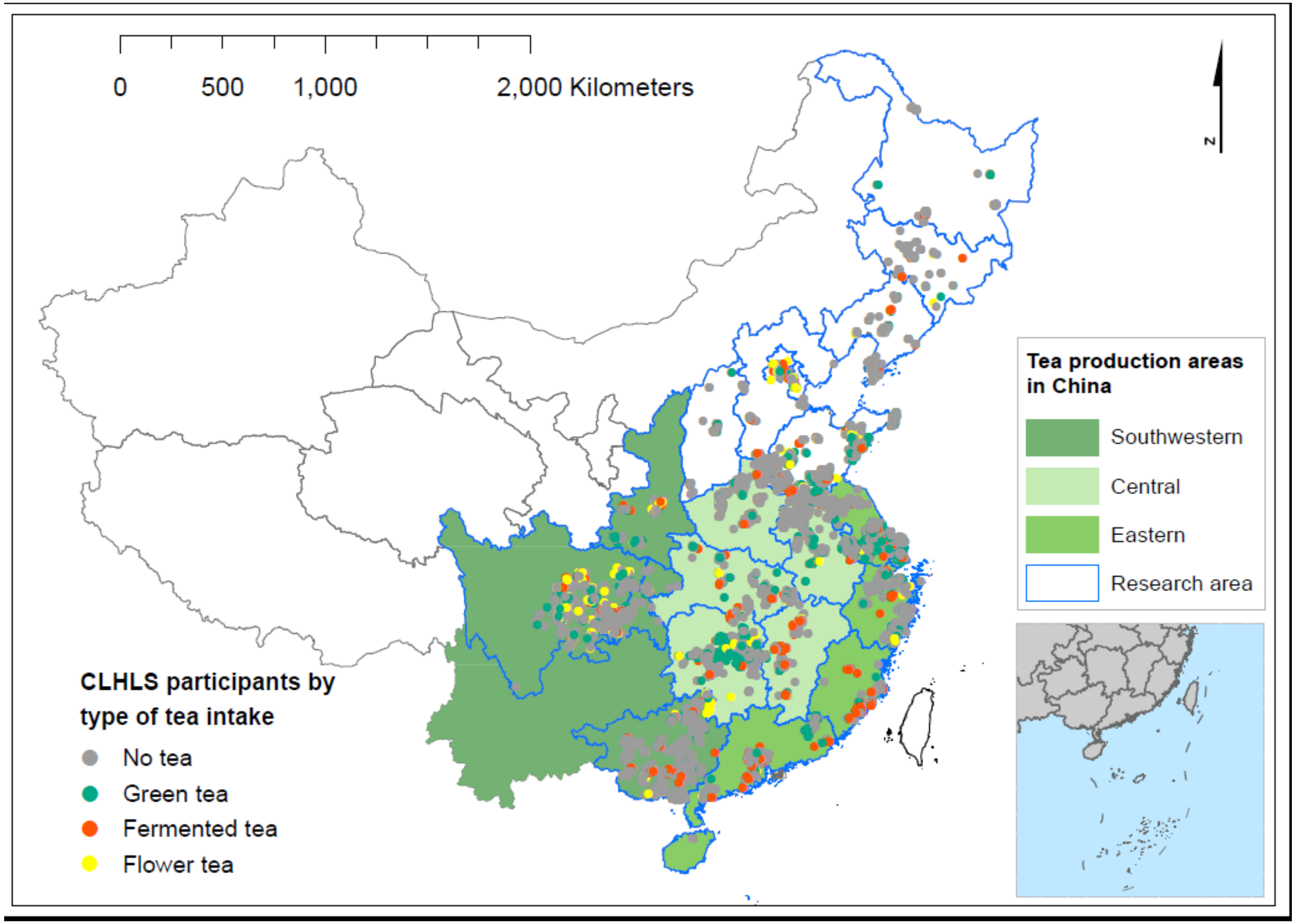

Note: Tea consumption was classified into No tea, Green tea, Fermented tea, and Flower tea, according to the self-reported habits of tea intake; the distribution of tea consumption was depicted according to the participants' resident location. The participants are from 23 provinces of China and they are shown in area of the blue frame. According to the Economic Regionalization Scheme of the National Bureau of Statistics of China, tea production regions were classified into Southwestern, Central, and Eastern areas. The map was made by the authors.

Supplementary Contents

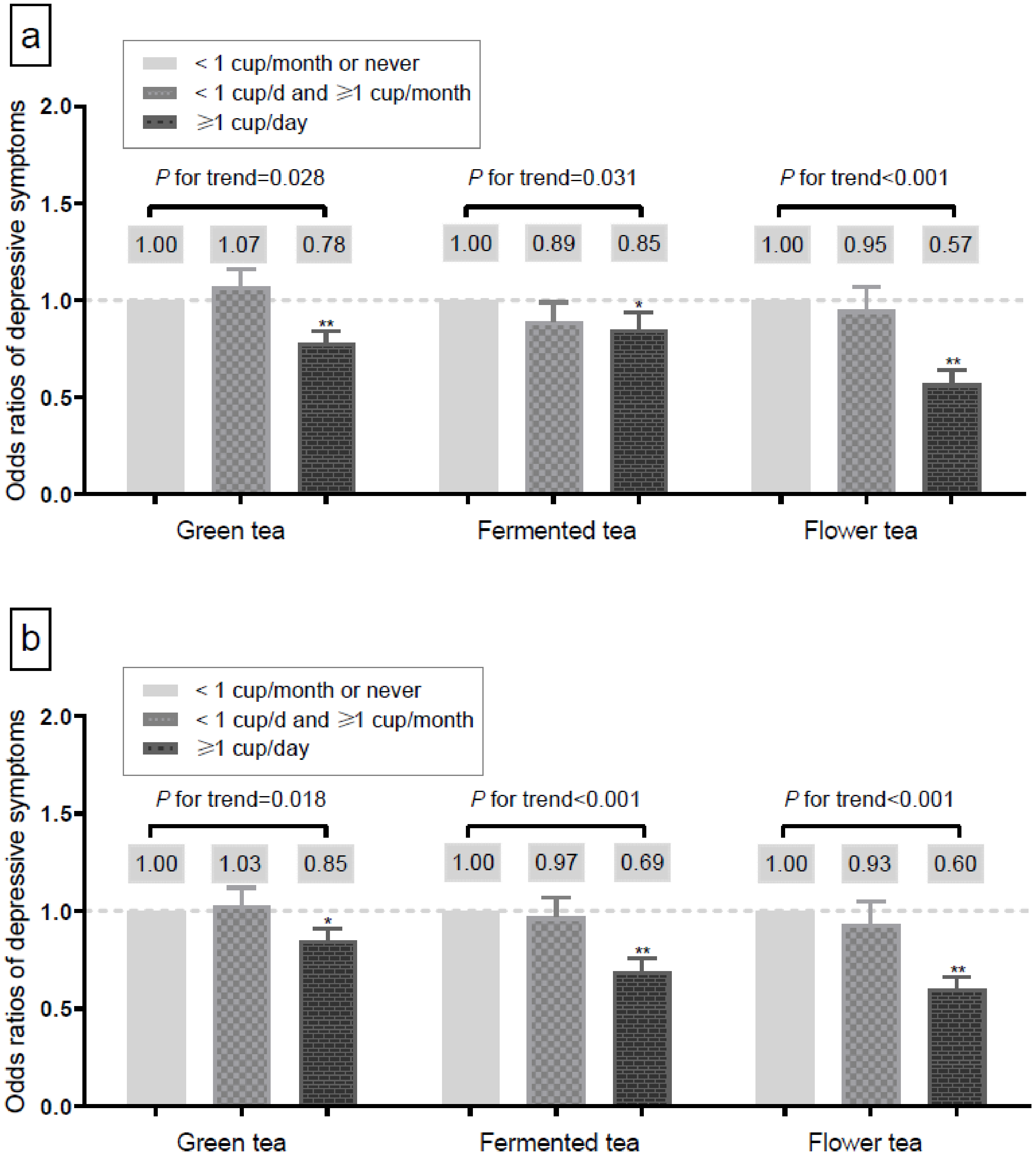

Supplementary Figure 2

Sensitivity analysis of type and frequency of tea intake with depressive symptoms by (a) using different cut-off value of CES-D-10=12 and (b) using different cut-off value of CES-D-10=8 as the definition of depressive symptoms. Main model included types of tea consumption as the sole variable and controlling for demographic and socioeconomic variables (age, gender, education, socioeconomic status, rural residence and geographical regions), psychosocial and behavioral variables (marital status, living arrangement, social and leisure activity index, smoking, alcohol drinking, BMI, regular dietary [vegetable/fruit/fish/nut] intake), and health variables (self-rated health, cognitive impairment, and medical illness, comorbidity, and ADL disability).

[*] P<0.05

[**] P<0.01

Supplementary Contents

Supplementary Figure 3

Sensitivity analysis of type and frequency of tea intake with depressive symptoms by (a) removing participants with severe cognitive impairment (MMSE<19; n=1,432) and (b) removing participants who were bedridden or terminally ill (n=261). Main model included types of tea consumption as the sole variable and controlling for demographic and socioeconomic variables (age, gender, education, socioeconomic status, rural residence and geographical regions), psychosocial and behavioral variables (marital status, living arrangement, social and leisure activity index, smoking, alcohol drinking, BMI, regular dietary [vegetable/fruit/fish/nut] intake), and health variables (self-rated health, cognitive impairment, and medical illness, comorbidity, and ADL disability).

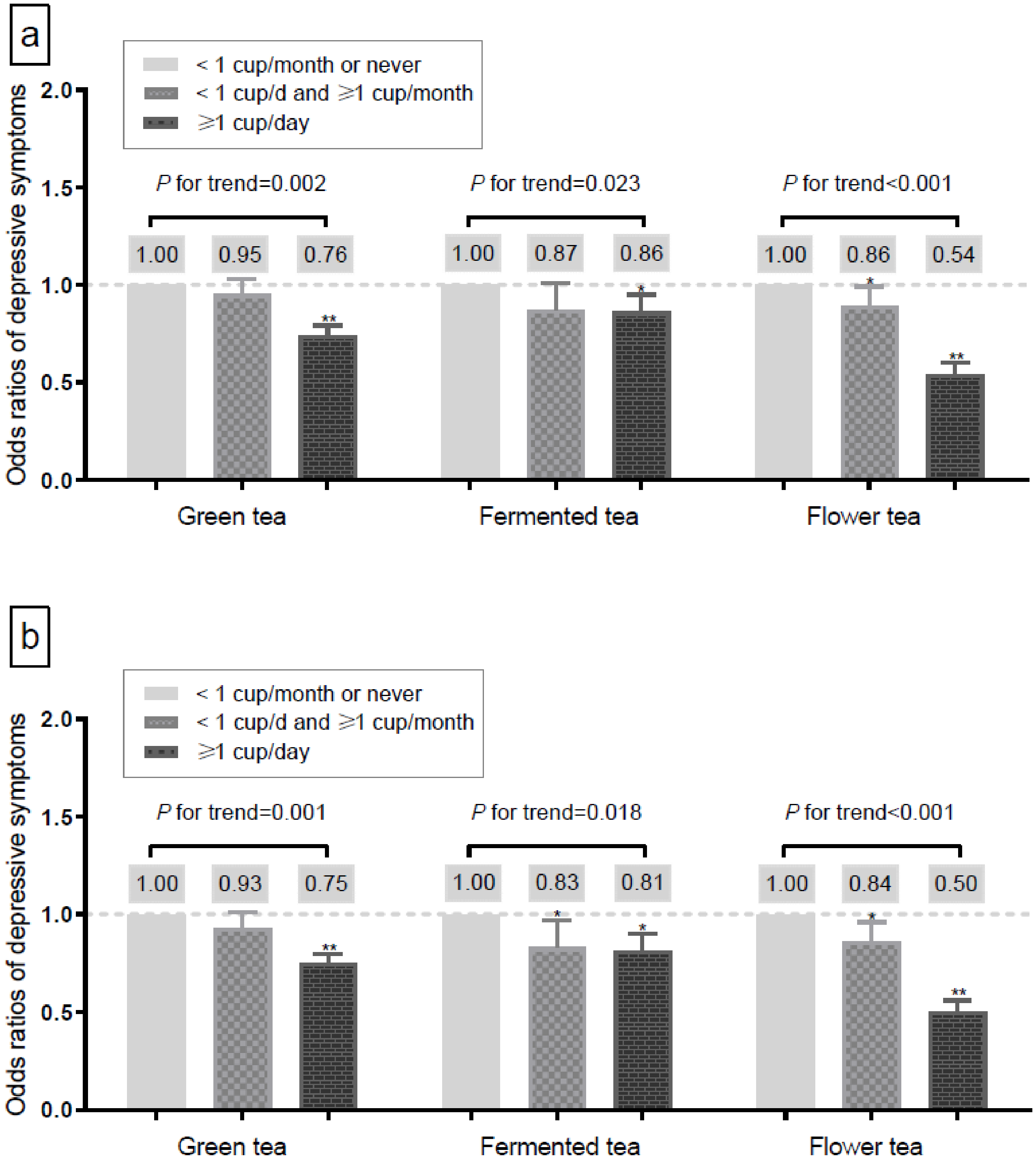

Note: * P<0.05; ** P<0.01

Supplementary Contents

Supplementary Figure 4

Sensitivity analysis of type and frequency of tea intake with depressive symptoms by (a) using full sample after multiple imputation (n=13,825) and (b) by adjusting sampling weight based on age-sex-residence-specific distribution of 2015 mini-census of China. Main model included types of tea consumption as the sole variable and controlling for demographic and socioeconomic variables (age, gender, education, socioeconomic status, rural residence and geographical regions), psychosocial and behavioral variables (marital status, living arrangement, social and leisure activity index, smoking, alcohol drinking, BMI, regular dietary [vegetable/fruit/fish/nut] intake), and health variables (self-rated health, cognitive impairment, and medical illness, comorbidity, and ADL disability).

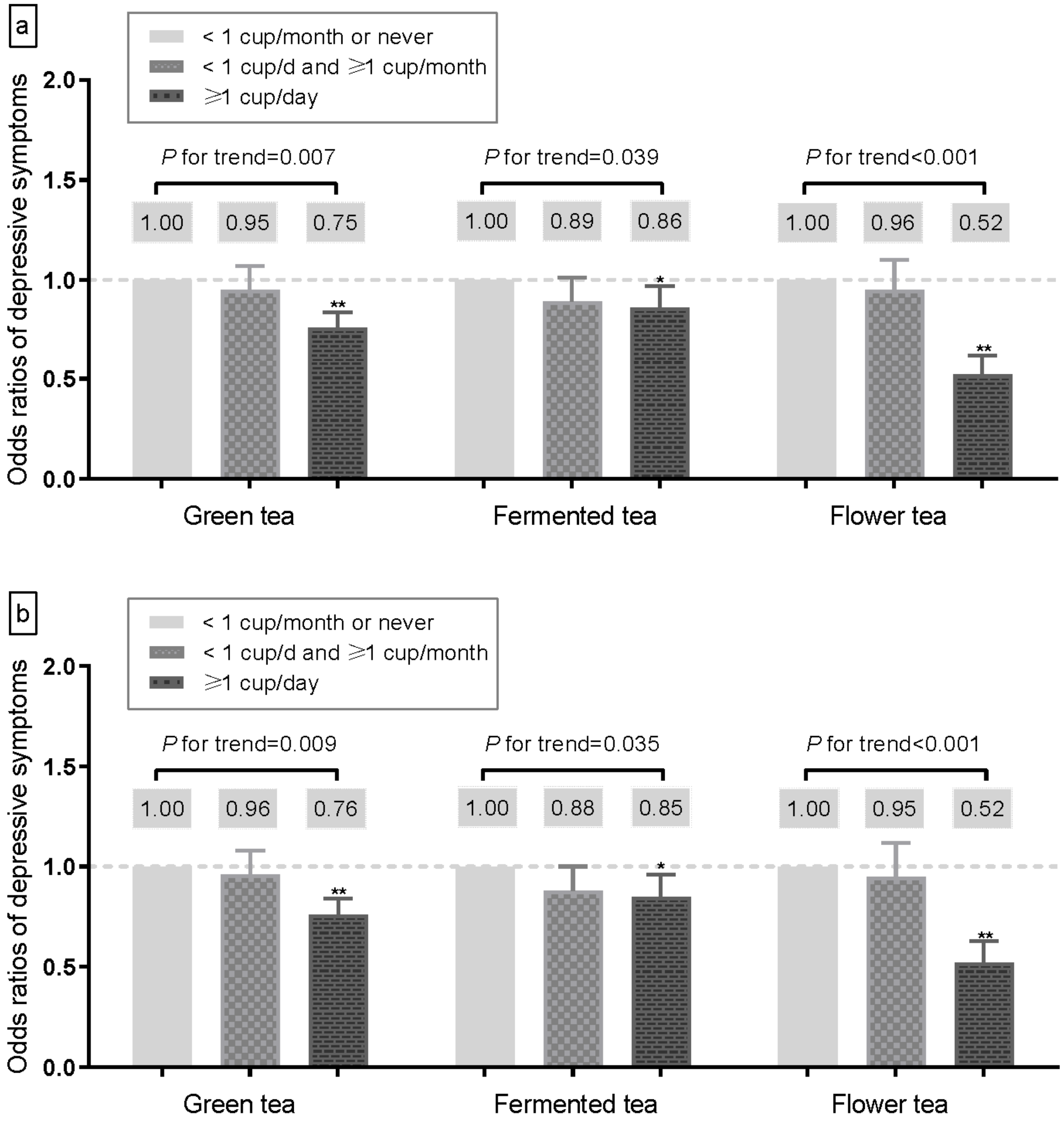

Note: * P<0.05 ** P<0.01
